# Leucine Regulates the Exocrine Function in Pancreatic Tissue of Dairy Goats In Vitro

**DOI:** 10.1155/2019/7521715

**Published:** 2019-10-13

**Authors:** Yangchun Cao, Kai Liu, Shimin Liu, Long Guo, Junhu Yao, Chuanjiang Cai

**Affiliations:** ^1^College of Animal Science and Technology, Northwest A&F University, Yangling 712100, China; ^2^UWA School of Agriculture and Environment, The University of Western Australia, Crawley, WA 6009, Australia

## Abstract

This study aimed to investigate the effects of leucine (Leu) on the synthesis and secretion of digestive enzymes in cultured pancreatic tissue of dairy goats and on the signaling molecules. Fresh pancreatic tissue from dairy goats was cut into approximately 2 mm × 2 mm pieces and incubated in oxygenated Krebs-Ringer bicarbonate buffer containing 0 (the control), 0.40, 0.80, or 1.60 mM Leu at 39°C in a CO_2_ incubator for 180 min. The results showed that Leu increased the release of *α*-amylase, trypsin, and chymotrypsin in the buffer and tissue, as well as the total activity (*P* < 0.05), especially at 0.40 and 0.80 mM. Compared with the control, 1.60 mM Leu increased the release of *α*-amylase and the total activity of trypsin and chymotrypsin (*P* < 0.05) but had no effect on the tissue concentration of *α*-amylase, trypsin, and chymotrypsin or the total activity of *α*-amylase (*P* > 0.05). Leu improved the mRNA expression of *α*-amylase, trypsin, and chymotrypsin (*P* < 0.05), especially at 0.80 and 1.60 mM. The activity and mRNA expression of lipase were not affected (*P* > 0.05). Compared with the control, 0.40 and 0.80 mM Leu increased the expression of the *γ* isoform of 4EBP1 (*P* < 0.05), implying increased phosphorylation of 4EBP1. Leu increased the phosphorylation of S6K1 (*P* < 0.05). Compared with the control, 0.40 and 0.80 mM Leu decreased the eEF2 phosphorylation level (*P* < 0.05). Conclusively, these results suggested that Leu could regulate the synthesis of pancreatic enzymes by increasing the mRNA expression and phosphorylation level of protein factors in the mammalian target of rapamycin pathway and the optimal Leu level in this experiment was 0.80 mM.

## 1. Introduction

The synthesis of proteins is completed by mRNA translation, which consists of initiation, elongation, and termination. The synthesis rate depends on the amount of mRNA and the intracellular ribosomes, as well as their translation efficiency [1]. The translation efficiency is dependent on the initiation and elongation of translation, which is regulated by eukaryotic initiation and elongation factors [2].

Mammalian target of rapamycin (mTOR) is a kinase, which serves as a core catalytic component of mTOR complex 1 (mTORC1) and mTOR complex 2; the mTOR pathway plays a very important role in the translation initiation and is the central regulator of metabolism of animal bodies [3, 4]. As a highly conserved protein, mTOR can sense cellular nutrient, oxygen, and energy levels [5]. It has been shown that the phosphorylation status of its upstream and downstream target protein molecules in the mTOR pathway can be regulated by amino acids, leading to stimulation of the protein synthesis [6]. Compared with nonessential amino acids, essential amino acids (3.5 mmol/l) increased mTOR phosphorylation by 100%, and the depletion of leucine (Leu) or isoleucine (Ile) alone could result in a decrease in mTOR phosphorylation by 57% or 47%, respectively [7, 8]. In addition, signal transmission by mTORC1 could be strongly inhibited by a lack of energy or amino acids, and a supply of amino acids to starving cells could significantly increase the activity of mTORC1 [9]. However, the molecular mechanism of regulation of the mTOR pathway by amino acids is still unclear.

Therefore, we hypothesized that Leu, as a functional amino acid, could change the phosphorylation status of the mTOR signal pathway, which could result in an increase in enzyme synthesis and excretion in the pancreas. To test this hypothesis, we focused on the main signal factors, including eukaryotic initiation factor 4E binding protein 1 (4EBP1), ribosomal protein S6 kinase 1 (S6K1), and eukaryotic elongation factor 2 (eEF2). The main goals of this study were to investigate the effects of Leu on the mTOR signal pathway and to define the associations between these signalling activities and the synthesis of pancreatic enzymes using an in vitro model of cultured pancreatic tissue of dairy goats.

## 2. Materials and Methods

All procedures used in this experiment complied with the animal care protocol that was approved by the Northwest A&F University Animal Care and Use Committee.

### 2.1. Pancreatic Tissue Preparation

Three one-year-old healthy Guanzhong dairy goats were used for taking pancreatic tissue. The three goats were slaughtered one goat per day over three days to provide fresh pancreatic tissues for the cultures.

When the goats were slaughtered, the caudal portion of the pancreas was removed immediately. The techniques described are based on similar procedures used for other species or purposes [10–16]. Briefly, mesentery, fat, and lymph were removed from the pancreas. Approximately 10 g of pancreas tissue from each goat was quickly excised once the goat was dead, placed in ice-cooled saline (0.9% NaCl), and immediately sent to the laboratory.

### 2.2. Tissue Isolation and Incubation

The incubation techniques described were based on the same procedures used in our previous study [17]. The pancreas piece was transferred to ice-cold Krebs-Ringer bicarbonate for tissues isolation and incubation. A separate aliquot of tissue was homogenised in saline and stored at −30°C until analysis of *α*-amylase, trypsin, and lipase activity.

### 2.3. Treatments and Experimental Design

There were four Leu treatments with medium containing 0, 0.40, 0.80, and 1.60 mM Leu. Dulbecco's modified Eagle's medium (DMEM)/Ham' F12 (HyClone, Logan, UT, USA) was used as the basal culture medium. The working Leu concentrations in the medium were 0.40, 0.80, and 1.60 mM Leu, and the control medium (0 mM) was the same as the treatment medium but contained no Leu (special custom medium; HyClone). After incubation for 180 mins, the tissues were harvested by scraping in the presence of ice-cold lysis buffer containing 1% (v : v) protease and phosphatase inhibitor cocktails (Roche, China). The culture medium was also collected for further analysis of enzymes activity. Culturing of the tissues was repeated for 3 days. On each day, tissues from a goat were cultured in four culture flasks, each containing one of the four kinds of media. Therefore, each Leu treatment had a total of three replicates from three goats (*n* = 3).

### 2.4. Sample Analysis

#### 2.4.1. In Vitro Enzyme Release

To measure the activity of digestive enzymes in the tissue segments, we collected the supernatant of the homogenate after homogenising. The activities of *α*-amylase, trypsin, and lipase in the supernatant of the homogenate and culture medium were determined using commercial kits (Nanjing Jiancheng Bioengineering Institute, China). One unit of enzyme activity was defined as 1 *μ*mol of product released per minute at 39°C.

#### 2.4.2. Quantification of Amylase, Trypsin, and Lipase mRNA Levels

RNA was isolated from the pancreas samples according to the method described by previous studies [17, 18]. The total RNA concentration was quantified by measuring its absorbance at 260 nm using a NanoDrop 2000 spectrophotometer (Thermo Scientific Inc. Wilmington, DE, USA). The sample integrity was determined from the ratio of absorbance at 260 nm to 280 nm, which was equal to or greater than 2.0 in all samples [19]. Reverse transcription (RT) of the mRNA was performed using a PrimeScript® RT Reagent Kit (TaKaRa Biotechnology Co., Ltd., Dalian, China). Primers used for the PCR are listed in Table 1. The mRNA levels of various tissues were assessed using the 2^−ΔΔCt^ method [20], and *β*-actin was used as the internal housekeeping gene control [21].

#### 2.4.3. Protein Immunoblot Analysis

Immunoblot analysis of 4EBP1, S6K1, and eEF2 protein was performed according to the method described by previous studies [17, 22]. A ChemiDOC XRS + imaging system (Bio-Rad, Germany) was used to visualise the target protein bands using an enhanced chemiluminescence reaction (ECL Kit, Bio-Rad, Cat. 1705061, CA, USA). Western blots were developed and quantified using Image J software (Windows version, National Institution of Health). When performing SDS-PAGE, the 4EBP1, S6K1, and eEF2 bands were separated into three subtypes, and the highly phosphorylated subtype (*γ*-subtype) moved the slowest. The proportion of *γ*-subtype relative to the total 4EBP1, S6K1 and eEF2 protein was calculated to indicate the extent of 4EBP1, S6K1, and eEF2 phosphorylation, respectively. The primary antibodies used were rabbit anti-goat polyclonal serum antibodies (4EBP1 from Calbiochem, Germany; S6K1 and eEF2 from Abcam, UK), and the secondary antibodies were goat anti-rabbit horseradish peroxidase conjugates (Beijing Synthetic Technology Co., Ltd., China).

### 2.5. Statistics

Analyses of enzyme activities, mRNA expression, and 4EBP1, S6K1, and eEF2 phosphorylation content were performed using the GLM procedure for a one-way analysis of variance (ANOVA) model in SAS software. Protein expression content was calculated relative to that of *β*-actin based on the band intensities. Differences with *P* < 0.05 were considered significant, and the data are presented as the mean ± standard error of the mean (SEM).

## 3. Results

As shown in Table 2, Leu increased the release of *α*-amylase, trypsin, and chymotrypsin into the buffer and tissue, as well as their total activities (*P* < 0.05), especially at concentrations of 0.40 and 0.80 mM. Compared with the control, 1.60 mM Leu increased the release of *α*-amylase and the total activity of trypsin and chymotrypsin (*P* < 0.05) but not the tissue concentrations of *α*-amylase, trypsin, and chymotrypsin or the total activity of *α*-amylase (*P* > 0.05). The activity of lipase was unaffected (*P* > 0.05).

**Figure 1:**
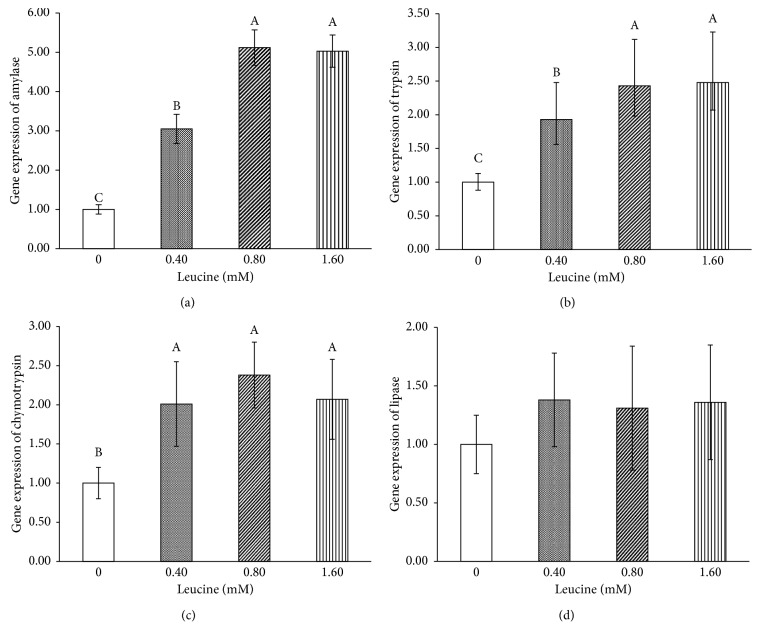
Effects of leucine on pancreatic amylase (a), trypsin (b), chymotrypsin (c), and lipase (d) mRNA levels in vitro. Values are the mean and pooled standard error of the mean (SEM). The enzyme mRNA levels were normalized to those of *β*-actin mRNA, and values of the mRNA levels were compared with those of the control group as 1.00. Different letters represent significantly different values (*P* < 0.05, *n* = 3).

As shown in Figure 1, Leu increased the mRNA expression of *α*-amylase, trypsin, and chymotrypsin (*P* < 0.05), especially at concentrations of 0.80 and 1.60 mM. The mRNA expression of lipase was unaffected (*P* > 0.05).

**Figure 2:**
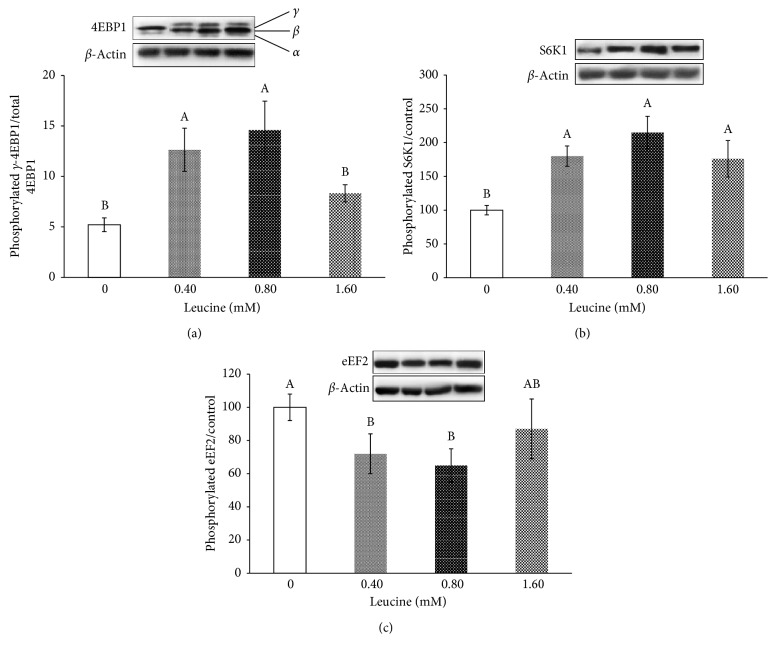
Effects of leucine on the ratio of phosphorylated to total mTOR signalling pathway factors in pancreas tissue. (a) 4EBP1 (*α*, *β*, and *γ* forms are denoted); (b) S6K1; (c) eEF2. The data are the means and pooled standard errors of the means (SEM). Different letters represent significantly different values (*P* < 0.05, *n* = 3).

Compared with the control, 0.40 and 0.80 mM Leu increased the expression of the *γ* isoform of 4EBP1 (Figure 2(a), *P* < 0.05), which means that the phosphorylation of 4EBP1 was increased. Leu increased the phosphorylation of S6K1 (Figure 2(b), *P* < 0.05). Compared with the control, 0.40 and 0.80 mM Leu decreased the eEF2 phosphorylation level (Figure 2(c), *P* < 0.05).

## 4. Discussion

The method of tissue incubation in vitro evaluating the regulation of pancreatic exocrine function in response to nutrients has rarely been studied, especially in ruminants, and there have only been three papers elaborating on this method in dairy cows [16, 17, 23]. This study improved on the in vitro tissue incubation method based on previous studies [10–16]. The key to successfully incubate tissues is to shorten the time involved in sampling and preprocessing of tissues, as well as the incubation conditions. A RS Biotech as a cell culture chamber rather than flasks filled with 95% O_2_ and 5% CO_2_ [16], as well as a thermostat concussion water bath tank, were used in this experiment, which ensured the accuracy of the experiment.

Three measurements of the activities of the enzymes were performed in this experiment: tissue concentration, release into the buffer, and total activity (the sum of the tissue concentration and release). The total activity indicates the synthesis of enzymes by the pancreas tissue, and the release of enzymes indicates the amount of enzyme that could be active for nutrient digestion. Leu increased the tissue concentration, release, and total activity of *α*-amylase, especially at 0.40 and 0.80 mM, while no effect on the activity of *α*-amylase was found at 1.60 mM compared with the control. A previous in vivo study in our laboratory showed that the concentration (U/ml) and the secretion rate (U/h) could be increased at most by 45.0% and 54.5%, respectively [24]. However, the total activity of *α*-amylase was increased by 24.7% at most in the present study, which is lower than the effect observed in animals [24]. A reason for this could be ascribed to the multiple factors that affect the activity of *α*-amylase in animals but not in vitro, such as nutrients, neuroregulation, and hormonal regulation [25, 26]. The release of *α*-amylase was increased by Leu, and the effects at 0.40 and 0.80 mM were better than those at 1.60 mM, in contrast to the results found by previous study [16]. Different physiological stages and treatments in the different experiments could be the reason for different results, since calves were infused with Leu [16].

The activities of trypsin and chymotrypsin were increased by Leu. The total activity of trypsin and chymotrypsin could be increased by 225.6% and 222.3%, respectively, which were much higher levels than those in a previous study on heifers [24]. When the in vitro tissue incubation method is used, most of the other factors that could affect the synthesis of enzymes are excluded and Leu remains as the only main factor regulating pancreatic exocrine function; therefore, its effects on the synthesis of enzymes should be much more distinct.

Leu could improve the synthesis and secretion of pancreatic enzymes by regulating the expression of various mRNAs. The mRNA expression of *α*-amylase, trypsin, and chymotrypsin was increased by Leu, especially at high levels. It has been shown that Leu could regulate the transcription and translation of islet cells and other cells through the mTOR pathway and other pathways [27–30]. The activity of pancreatic *α*-amylase decreased with increasing starch flow in the duodenum, while protein administration could increase the secretion of *α*-amylase [16]. A previous study in dairy goats by our group showed that a high level (9 g/d) but not a low level (3 g/d) of Leu increased the mRNA expression of *α*-amylase, while no effects on the activities of trypsin and lipase were observed [31]. At 1.60 mM, Leu increased the mRNA expression of *α*-amylase, trypsin, and chymotrypsin but not the tissue concentrations of these enzymes.

Cells could change their gene expression profiles in response to different environments. Although proteins are the products of new mRNAs in response to the transcription stage, cells could also regulate gene expression by regulating translation [32–35]. Thus, as a preprocessing stage of protein translation, mRNA translation into proteins is largely but not crucially regulated by nutrients. The reason that high-level Leu (1.60 mM) could increase mRNA expression but not enzyme secretion could be related to the protein translation efficiency.

Previous studies have shown that branched-chain amino acids could regulate the protein synthesis of islet *β*-cells by activating the mTOR pathway [28, 29, 36, 37]. It has been verified that 0.4–4.0 mM Leu could increase the phosphorylation level of p70S6K in islet *β*-cells which increases protein synthesis, and this process is sensitive to rapamycin but not insulin [28, 37, 38]. A study using the mammary epithelial cells of cows showed that the deprivation of all amino acids or Leu alone could decrease the phosphorylation of 4EBP1 and S6K1 in the mTOR pathway [39]. In addition, the administration of Leu alone could increase the phosphorylation level of mTOR as well as the synthesis rate of protein fragments [7, 8].

In our experiment, Leu could increase the phosphorylation level of 4EBP1 and S6K1 and decrease the phosphorylation of eEF2, which is in accordance with a previous study by our team [31]. In terms of the effects of Leu on eEF2, previous studies have shown that the activity of eEF2 increased linearly with the levels of essential amino acids but not with Leu alone [7, 8]. The reason for this finding could be related to the different experimental methods. A cell starvation method was used in the experiment in which antagonistic amino acids were applied that weakened or counteracted the effects of Leu on eEF2 [8]. In addition, the regulation of protein factors in the translation process increased first and then decreased with a peak at 0.80 mM Leu, which is consistent with the results by Apelo et al. [40].

## 5. Conclusion

In conclusion, Leu could regulate the synthesis of pancreatic enzymes by increasing the mRNA expression and the phosphorylation level of protein factors in the mTOR pathway, and the optimal level of Leu in this experiment was 0.80 mM.

## Figures and Tables

**Table 1:** Primer sequences.

Target genes	Reference sequence	Primer sequence (5′-3′)	Product size (bp)	Annealing temperature (°C)
Amylase	NM_001035016	F: GAAATGGCCGTGTGACAGAATTTA	142	64.3
		R: ACAAAGACAAGTGCCCTGTCAGAA		
Trypsin	NM_001113727	F: TGTCTGCGGCTCACTGCTAC	119	62.7
		R: GCTGGGATGGACGATACTCTTG		
Chymotrypsin	NM_001098965.1	F: ATGTTGGGCATCACGGTCTT	172	60.0
		R: TGTGCCTCCACGTGTTATCC		
Lipase	NM_001205820	F: GTGGAAGCAAATGATGGACAAG	81	61.8
		R: TGGGTTGAGGGTGAGCAGA		
*β*-Actin	AF481159	F: ACCACTGGCATTGTCATGGACTCT	152	60.0
		R: TCCTTGATGTCACGGACGATTTCC		

**Table 2:** Effects of leucine on the activities of enzymes in vitro.

Item	Levels of leucine (mM)^1^				SEM^2^	*P*
	0	0.40	0.80	1.60		
Tissue concentration (U/g)						
*α*-Amylase	572^b^	681^a^	658^a^	581^b^	48.5	0.017
Trypsin	3.45^c^	5.69^b^	7.67^a^	3.88^c^	0.573	<0.001
Chymotrypsin	2.68^b^	4.31^a^	4.86^a^	3.11^b^	0.736	0.008
Lipase	402	497	530	525	38.8	0.258
Release (U/g tissue)						
*α*-Amylase	221^c^	308^a^	310^a^	268^b^	37.6	0.012
Trypsin	1.05^b^	2.57^a^	2.48^a^	2.71^a^	0.474	0.029
Chymotrypsin	0.88^b^	2.30^ab^	2.61^a^	2.77^a^	0.385	0.024
Lipase	116	112	125	121	17.1	0.207
Total activity (U/g tissue)						
*α*-Amylase	793^b^	989^a^	968^a^	849^b^	75.145	<0.001
Trypsin	4.50^c^	8.26^ab^	10.15^a^	6.59^b^	0.652	0.005
Chymotrypsin	3.36^c^	6.61^ab^	7.47^a^	5.88^b^	0.536	0.012
Lipase	518	607	655	646	20.7	0.092

^1^One-way ANOVA. Differences were considered significant at *P* < 0.05. ^2^Pooled standard error of the means, *n* = 3, ^a–c^Means within a row not sharing the same superscript differ significantly (*P* < 0.05).

## Data Availability

The data used to support the findings of this study are available from the corresponding author upon request.
